# Miliary brain metastasis presenting with calcification in a patient with lung cancer: a case report

**DOI:** 10.1186/1752-1947-6-279

**Published:** 2012-09-04

**Authors:** Minehiko Inomata, Ryuji Hayashi, Kenta Kambara, Seisuke Okazawa, Shingo Imanishi, Tomomi Ichikawa, Kensuke Suzuki, Toru Yamada, Toshiro Miwa, Tatsuhiko Kashii, Shoko Matsui, Kazuyuki Tobe, Masakiyo Sasahara

**Affiliations:** 1 First Department of Internal Medicine, University of Toyama, Toyama City, 930-0194, Japan; 2 Department of Medical Oncology, Toyama University Hospital, Toyama City, 930-0194, Japan; 3 Health Administration Center, University of Toyama, Toyama City, 930-0194, Japan; 4 Department of Pathology, University of Toyama, Sugitani 2630, Toyama City, 930-0194, Japan

## Abstract

**Introduction:**

Miliary brain metastasis is an extremely rare form of brain metastasis which can present with atypical imaging findings. We report the case of a patient with miliary brain metastasis of lung cancer showing calcification in metastatic lesions.

**Case presentation:**

A 68-year-old Japanese woman was diagnosed with lung adenocarcinoma. Brain computed tomography revealed multiple small calcified lesions in both cerebral hemispheres. Mutation of the epidermal growth factor receptor gene (exon 21, L858R) in lung cancer cells was detected, and treatment with gefitinib was initiated. A partial response was observed; however, the patient was readmitted to our hospital because of regrowth of the primary lesion and complaints of nausea, headache, and difficulty walking. Brain magnetic resonance imaging revealed scattered tiny nodules enhanced by gadolinium. A diagnosis of leptomeningeal carcinomatosis was made on the basis of cerebrospinal fluid cytology. The patient’s general status worsened, and she died 356 days after the day of first medical examination. Upon autopsy, the brain was found to be edematous and swollen. Lung carcinoma cells were diffusely disseminated on the meningeal surface. Metastatic foci of small nodular form, accompanied by calcifications, were also found in the brain parenchyma. We diagnosed miliary metastasis of lung carcinoma.

**Conclusions:**

To the best of our knowledge, this is the third report of calcified miliary brain metastasis confirmed by autopsy. We describe calcified lesions that increased in size during the clinical course of nine months. Brain computed tomography findings that reveal multiple small calcified lesions in patients with malignancy should raise suspicion of miliary brain metastasis.

## Introduction

Miliary brain metastasis, which was reported by Madow and Alpers in 1951 as carcinomatous encephalitis, is a rare form of brain metastasis [1]. It is sometimes difficult to diagnose, owing to atypical imaging findings. We report the case of a patient with miliary brain metastasis of lung cancer showing calcification in metastatic lesions.

## Case presentation

**Figure 1:**
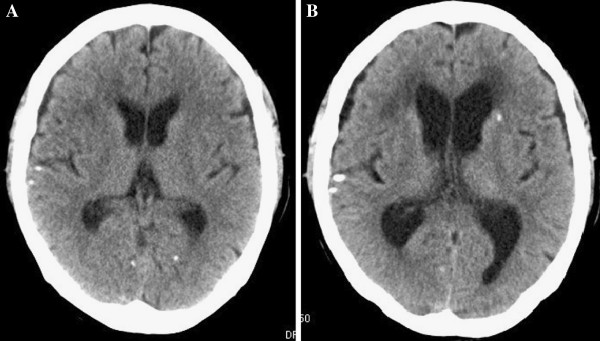
Brain computed tomography imaging without intravenous contrast (A) and (B) showed tiny calcified lesions in both cerebral hemispheres (A and B) and enlarged lateral ventricles (B).

**Figure 2:**
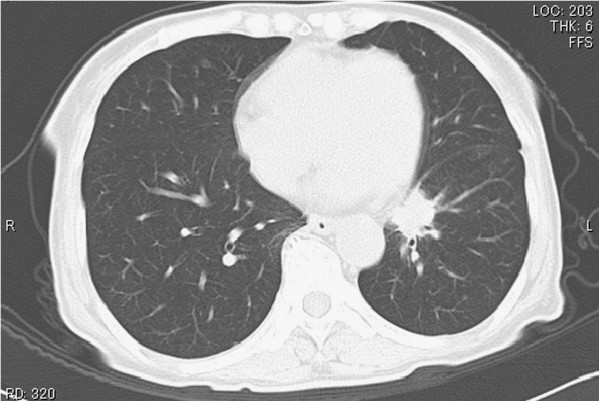
Chest computed tomography imaging on admission showed the primary lesion in the left lower lobe.

**Figure 3:**
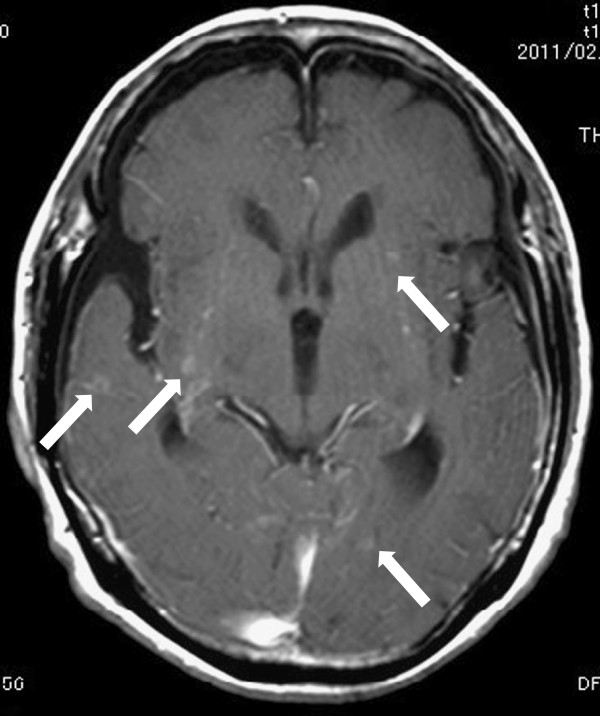
T1-weighted gadolinium contrast-enhanced magnetic resonance imaging showed multiple enhanced tiny nodules (arrows).

A 68-year-old Japanese woman complained of pain in the right upper arm, and a diagnosis of humerus fracture was made at a department of orthopedics in a local general hospital. Adenocarcinoma was detected by biopsy performed during surgery. Lung cancer was suspected on the basis of the findings of chest computed tomography (CT) showing a tumor in the left lower lobe of the lung, and the patient was referred to our hospital in April 2010. After further examinations, including transbronchial biopsy of the tumor in the left lower lobe, the patient was diagnosed as having advanced lung adenocarcinoma (T3N2M1b) with mutation of the epidermal growth factor receptor gene (exon 21, L858R). Brain CT revealed scattered, small calcifications in both hemispheres (Figure 1A), but no neurological abnormality was seen. The patient was treated with gefitinib, and a partial response was obtained 14 days after the initiation of treatment. However, the primary lesion showed regrowth at 263 days after the initiation of treatment, and the patient complained of nausea, headache, and difficulty walking and was readmitted to our hospital. Unsteadiness of gait was observed. Hematological and biochemical examinations did not detect any abnormal findings. Chest CT showed the primary lesion in the left lower lobe, which had not enlarged much since the first examination (Figure 2). In brain CT without intravenous contrast, the calcified lesion was shown to have slightly increased in size, and enlarged lateral ventricles were seen (Figure 1B). T1-weighted gadolinium contrast-enhanced magnetic resonance imaging (MRI) revealed multiple enhanced tiny nodules (Figure 3).

On the basis of these findings, multiple brain metastases or leptomeningeal carcinomatosis was suspected, and lumbar puncture was performed. Atypical cells were detected, and we diagnosed leptomeningeal carcinomatosis of lung cancer. However, the patient’s general status worsened, and she died 356 days after the first medical examination at our hospital. Upon autopsy, a nodular lesion 2cm in diameter was detected in the lower lobe of the left lung. Adenocarcinoma of combined acinar and bronchioalveolar type was microscopically diagnosed, which was accompanied by microcalcification. The brain was swollen and edematous. The meninges were cloudy. On the cut surface, the third and fourth ventricles were dilated. Nodular lesions smaller than 6mm in diameter were detected in the brain parenchyma, including the cerebral cortex and basal ganglia. On microscopic examination, minute metastatic foci of adenocarcinoma that were extensively spread showed close cell morphologies with lung carcinoma. Microscopically, carcinoma cells were extensively disseminated, without formation of mass lesions, on the meninges from the cerebral cortex to the brain stem.

**Figure 4:**
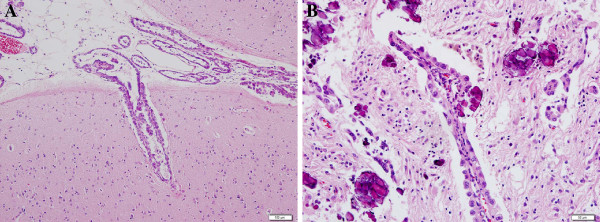
**Histopathological specimens showed adenocarcinoma mainly distributed in perivascular spaces of Virchow–Robin, without tumoral mass or surrounding edema (A).** Calcifications were also detected (**B**).

In accordance with the distribution of these lesions, the carcinoma cells frequently invaded into the Virchow–Robin spaces in the superficial brain parenchyma. In a certain region, the carcinoma cells on meninges had spread, in a continuum, into a superficial part of the brain parenchyma via Virchow–Robin spaces. In addition, nodular metastatic foci were detected in the brain parenchyma, including cerebral cortex, thalamus, and cerebellar hemisphere, in accordance with the macroscopic findings. These nodular lesions were accompanied by microcalcification, and the tumor cells were often seen to be arranged in perivascular space within these lesions (Figure 4). Tiny foci of metastasis were detected in the pineal body and in the choroid plexus of the third and fourth ventricles as well. Mass effects of the metastatic lesions were insignificant, and edematous change was not apparent at the metastatic foci. However, brain parenchyma was edematous in general, with wide perivascular spaces, which was probably due to the disturbed flow of cerebrospinal fluid after disseminated metastasis. We diagnosed miliary brain metastasis of lung cancer based on these findings of imaging and histopathological examination.

## Discussion

The patient described herein presented an atypical clinical course and imaging findings for metastatic brain tumor of lung cancer. Twenty-two cases with miliary brain metastasis have been reported [1-15], and this is the third report of miliary brain metastasis with calcification, which was confirmed by autopsy [2,3]. Furthermore, we observed that calcified lesions increased in size during the clinical course of nine months.

The majority of previously described cases of miliary brain metastasis were from lung adenocarcinoma [1,2,4-10]. Miliary brain metastasis is diagnosed on the basis of findings showing diffuse miliary spread of punctate tumor nodules in the brain. Tumor cells are distributed in perivascular spaces without surrounding edema or mass effects [1,6-12], and it is suggested that tumor cells extend through the perivascular spaces into subpial surface [9]. There are no precise diagnostic criteria for miliary brain metastasis, however, and the size of metastatic lesions in cases previously reported ranges widely. The pathological findings obtained in the present case were similar to those used to diagnose miliary brain metastasis previously.

Imaging examinations in the majority of reported cases showed numerous tiny nodules in the cerebral hemispheres, reflecting pathological findings. However, 4 of 17 cases in which brain MRI was performed did not show any abnormalities [6,7,9,13], and four of 13 cases in which gadolinium-enhanced brain MRI was performed did not show any contrast effects [4,6,11,13]. It is speculated that contrast-enhanced MRI could fail to delineate the metastatic lesions because the blood–brain barrier remains intact at an early point in the clinical course [9,11]. Nemzek *et al*. reported a case in which T2-weighted MRI detected punctate lesions, even though there were no contrast effects [11]; however, the findings were not typical for brain metastatic tumors. In our case, there could have been more metastatic lesions than we visualized by brain CT initially. Four of 22 (18.2%) cases with miliary brain metastasis (including the present case) showed calcified lesions [2-4]. Miliary brain metastasis appears to exhibit calcified lesions more frequently than all cases of metastatic brain tumor, in which calcification is reported to be shown in 3.5%. The mechanism of calcification of miliary brain metastasis has not been fully understood. However, four mechanisms of calcification within lung cancer have been suggested: (1) calcified scar tissue or granulomatous disease engulfed by tumor, (2) dystrophic calcification within areas of tumor necrosis, (3) calcium deposition within the tumor as a result of the secretory function of the carcinoma itself, and (4) metastatic calcification from an elevated level of calcium phosphate product [14]. In our case, calcification occurred in primary tumor and miliary brain metastatic lesions. It was deposited within cancer cells. The histological type of this tumor was adenocarcinoma of combined acinar and bronchoalveolar type. These findings suggest that calcification resulted from the secretory function of the carcinoma itself (item (3) above) [15].

The patient described herein initially had no symptoms of brain metastasis and subsequently complained of headache and nausea due to leptomeningeal carcinomatosis. In previous cases, convulsion [1,13], hemiparesis [1,10], memory disturbance [7-9], dementia-like symptoms or psychiatric symptoms [1-4,8-11,13,16,17], and disorientation [5-9,16] have been reported as primary symptoms. Patients with miliary brain metastasis can show an atypical clinical course and atypical symptoms.

The therapeutic strategy for miliary brain metastasis has not been established, owing to the rarity of this disease. Radiotherapy for miliary brain metastasis showed a response in only one of three reported cases [12,16,17]. Another patient who received treatment with radiation and administration of gefitinib showed complete resolution of brain metastases [5]. It is necessary to accumulate cases to establish an effective therapy for this rare condition.

## Conclusions

In summary, patients with miliary brain metastasis are sometimes difficult to diagnose, owing to atypical imaging findings, such as scattered small, calcified lesions. Abnormality of neurological findings, psychiatric symptoms, or worsened general status shown in patients with malignancy, which is difficult to explain by imaging findings, should raise suspicion about the possibility of this disease entity.

## Consent

Written informed consent was obtained from the patient’s next of kin for publication of this manuscript and accompanying images. A copy of the written consent form is available for review by the Editor-in-Chief of this journal.

## Competing interests

The authors declare that they have no competing interests.

## Authors’ contributions

MI, RH, KK, SO, TI, KS, and KT were involved in treatment of the patient. TY, TM, TK, and SM interpreted the patient data and were involved in treatment planning. MI, RH, and SI were involved in preparing the manuscript. MS performed histological examination. All authors read and approved the final manuscript.
